# High-intensity exercise increases breast milk adiponectin concentrations: a randomised cross-over study

**DOI:** 10.3389/fnut.2023.1275508

**Published:** 2023-12-18

**Authors:** Mads Holmen, Guro F. Giskeødegård, Trine Moholdt

**Affiliations:** ^1^Department of Circulation and Medical Imaging, Norwegian University of Science and Technology, Trondheim, Norway; ^2^K.G. Jebsen Center for Genetic Epidemiology, Department of Public Health and Nursing, Norwegian University of Science and Technology, Trondheim, Norway; ^3^Women’s Clinic, St.Olavs Hospital, Trondheim, Norway

**Keywords:** high-intensity interval training, lactation, nutrition, adipokine, running, obesity

## Abstract

**Introduction:**

Adiponectin plays a role in glucose and fat metabolism and is present in human breast milk. It has been postulated that higher breast milk adiponectin concentrations may prevent rapid weight gain in infancy. Prior research indicates that circulating adiponectin increases acutely after endurance exercise, but no prior research has investigated the effect of exercise on breast milk adiponectin concentrations. The purpose of this randomised, cross-over study was to determine the acute effects of endurance exercise on adiponectin concentrations in human breast milk.

**Methods:**

Participants who were exclusively breastfeeding a 6–12 week-old term infant (*N* = 20) completed three conditions in the laboratory: (1) Moderate-intensity continuous training (MICT), (2) High-intensity interval training (HIIT), and (3) No activity (REST). At each condition, we collected breast milk at 07:00 h (before exercise/rest), 11:00 h (immediately after exercise/rest), 12:00 h (1 h after exercise/rest), and 15:00 h (4 h after exercise/rest) and determined adiponectin concentrations using enzyme-linked immunosorbent assay. We compared changes in adiponectin concentrations after MICT and HIIT, adjusted for the morning concentration on each test day, with those after REST, using paired *t*-tests.

**Results:**

Adiponectin concentrations increased 1 h after HIIT, from 4.6 (± 2.2) μg/L in the 07:00 h sample to 5.6 (± 2.6) μg/L. This change was 0.9 μg/L (95% confidence interval 0.3 to 1.5) greater than the change between these two timepoints in the REST condition (*p* = 0.025). There were no other statistically significant changes in adiponectin concentrations.

**Conclusion:**

HIIT may increase adiponectin concentrations in breast milk acutely after exercise. Further studies should determine the impact of exercise-induced elevations in breast milk adiponectin concentrations on growth and metabolism in infancy.

## 1 Introduction

The World Health Organization (WHO) estimated in 2020 that 39 million children under the age of 5 were overweight/obese, and that the prevalence of overweight/obesity among children and adolescents aged 5–19 years rose from 4% in 1975 to 18% in 2018 (1). One contributing factor for the rapid increase in childhood obesity is early nutritional programming: that nutrition in early life partly determines later health. Indeed, the period from conception to 2 years of age, often referred to as “the first 1000 days,” is the most critical period for pathophysiological disorders leading up to childhood and later life obesity (2). The mechanisms behind nutritional programming are not fully understood, but potentially include appetite regulation, epigenetic modifications, and changes in the gut microbiome (3–5). One of the reasons why WHO recommends exclusive breastfeeding for the first 6 months of life is that breastfed children have lower likelihood of becoming overweight/obese compared with bottle-fed children (6). However, recent evidence suggests that breast milk concentrations of nutrients and bio-active molecules vary between mothers with high and low body mass index (BMI), and that differences in breast milk composition may play a role in the mother-to-child transmission of obesity through lactational programming (7). Based on recent evidence, we have postulated that exercise may improve breast milk composition and thereby reduce the intergenerational transmission of obesity (8).

Breast milk contains adiponectin, an adipokine that plays a role in glucose and fat metabolism (9), which can cross the intestinal barrier and may modify infant metabolism (10). Adiponectin is a protein hormone mostly secreted into circulation from adipocytes in white adipose tissue (11). Low levels of circulating adiponectin are associated with insulin resistance and type 2 diabetes (12, 13). In 2006, adiponectin was shown to be present in human milk (14). Given the importance of adiponectin in inflammation, insulin sensitivity, and fatty acid metabolism, later studies have examined whether breast milk adiponectin has a role in infant metabolic development with a postulated protective effect of breast milk adiponectin on rapid weight gain in infancy (15). However, research on the association between breast milk concentration of adiponectin and measures of infant adiposity and weight gain has shown contrasting results, with some evidence suggesting an inverse association between levels of this hormone and infant adiposity measures (15–18), others showing no association (19–22), others yet a positive association (23–25).

Maternal lifestyle, including smoking, BMI, gestational diabetes, and diet, has been shown to affect the composition of breast milk, and this interplay with infant health is an area of research that is gaining traction (26–30). Exercise is a behavioural factor that has received little attention in this context. Aerobic exercise, either as moderate-intensity continuous training (MICT) or high-intensity interval training (HIIT), can increase circulating levels of adiponectin (31, 32). No prior study has investigated the effect of exercise on breast milk concentrations of adiponectin. The aim of this study was to determine the acute effect of one bout of MICT and HIIT on breast milk adiponectin concentrations. We hypothesised that both MICT and HIIT would increase adiponectin concentrations compared with no exercise.

## 2 Materials and methods

### 2.1 Study design and participants

**FIGURE 1:**
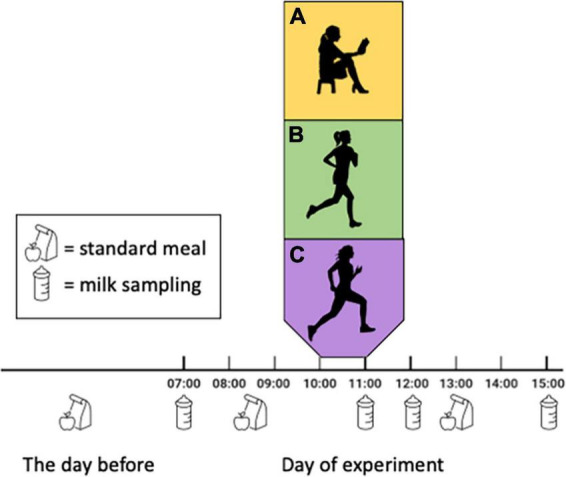
Study design. Participants completed three conditions in the laboratory, in random order: **(A)** sitting (REST), **(B)** moderate-intensity continuous training (MICT), and **(C)** high-intensity interval training (HIIT). Breast milk samples were collected at standardised timepoints in all conditions and conditions were separated by >48 h. We did not control for or record breastfeeding on test days.

This was a randomised cross-over study conducted at the Norwegian University of Science and Technology in Trondheim (NTNU), Norway. The Regional Committee for Medical and Health Research Ethics, Central Norway approved the study (REK-263493). The study was pre-registered in clinicaltrials.gov (NCT05042414, 13/09/2021). We included females aged 18 years or more who had given birth to a singleton, term infant 6–12 weeks ago. To be eligible, they had to exclusively breastfeed, be able to walk or run on a treadmill for at least 50 min, and live in the Trondheim area. Exclusion criteria were known cardiovascular disease or diabetes mellitus type 1 or 2. The participants signed an informed, written consent. In random order, the participants underwent three conditions: REST (sitting), MICT, and HIIT, with minimum 48 h washout between conditions (Figure 1). Randomisation was completed on the first test day. We used a computer random number generator developed at the Faculty of Medicine and Health Science, NTNU, to randomise the sequence of the conditions for each participant. The sequence was shown on the screen and sent by e-mail to the investigators. We did not inform the participants about which condition they were going to complete before they attended the laboratory on test days. Participants and investigators were not blinded due to the nature of the intervention (exercise). All methods were performed in accordance with the relevant guidelines and regulations.

### 2.2 Baseline assessments

The participants came in for assessments of body composition and peak oxygen uptake, on a separate day prior to the three conditions. We estimated body composition using bioimpedance (InBody720 Biospace Co., Republic of Korea). The participants completed a maximal effort exercise test on a treadmill, and we measured maximal oxygen uptake using MetaLyzer II with MetaSoft Software (CORTEX Biophysik, Germany). They wore heart rate monitors (Polar, Finland) during the test, and we used the highest heart rate during the test as an estimate of heart rate maximum (33). The participants also filled out questionnaires about background characteristics and physical activity levels (International Physical Activity Questionnaire) on this day.

### 2.3 Exercise and rest conditions

We requested the participants to abstain from exercise >48 h prior to all assessments in the laboratory. To minimise the effect of diet on breast milk composition, the participants recorded their diet (type of food, amount, and time of consumption) the evening prior to the first test day, as well as on the day of assessments (Figure 1). We requested them to repeat the same dietary intake for the two latter conditions.

For the REST condition, the participants rested in a chair in the laboratory for 45 min. We fitted the participants with heart rate monitors prior to the exercise conditions. Exercise intensity was based on percentage of heart rate maximum and the two protocols are isoenergetic (34). MICT consisted of walking or jogging for 48 min at 70% of heart rate maximum. In the HIIT condition, a 10-min warm-up at moderate intensity was followed by four 4-min work-bouts at 90–95% of heart rate maximum, separated by 3 min of low-to-moderate intensity. To calculate the actual exercise intensity during MICT, we recorded heart rate every fifth minute, whereas we recorded average heart rate in the last 2 min of every work-bout during HIIT.

### 2.4 Breast milk sampling

We provided the participants with electronic breast milk pumps (Medela Swing Flex, Medela AG, Switzerland). The participants sampled breast milk at four standardised timepoints in each of the three conditions: 07:00 h (before breakfast), 11:00 h (immediately after exercise/rest), 12:00 h (1 h after exercise/rest), and 15:00 h (4 h after exercise/rest) (Figure 1). The participants sampled from the same breast at each timepoint, and we asked them to provide at least 25 mL each time. We did not ask the participants to pump as much as possible, only to provide minimum 25 mL at each time point, before feeding their infant. The first (07:00 h) and last (15:00 h) samples were stored in the participants home freezer and transported on ice to the laboratory at the next visit, whereas the other samples were collected when the participants were in the laboratory. All samples were stored at −80° until analysis. We did not control for or record breastfeeding of the infant on the test days.

### 2.5 Adiponectin analysis

After thawing the breast milk at room temperature, we centrifuged samples at 10,000 × *g* for 60 min. The fat layer on the top was carefully removed using tweezers and skimmed milk extracted for analysis. We used enzyme-linked immunosorbent assay (ELISA) for quantitative measurement of adiponectin (IBL International GmBH, Germany, Catalog no: 30126762), using a Dynex DS2 automation system programmed with compatible DS-Matrix software (Montebello Diagnostics AS, Norway). The intra- assay variability for the ELISA kit is <5% and inter-assay variability 7.5%. Undiluted breast milk samples were measured in duplicate wells with samples from the same participant on the same microtitre plate (using the same kit). The coefficient of variability for the duplicates was 2.0 (SD 1.5). Based on our pilot testing of the ELISA kits, we set the time for the final incubation step to 12 min, instead of 15 min as described in the instruction manual, otherwise we followed the manufacturer’s instruction. The range of the ELISA assay was 0.27–31000 μg/L and all measurements were obtained in the linear range of the assay. The coefficient of determination for the standard curve was 1.

### 2.6 Statistical analysis

No formal sample size calculation could be done for this study due to the exploratory nature of the research question. We aimed to recruit 20 participants. Crossover studies allows comparison at the individual rather than the group level and fewer participants are required in a crossover design compared with a parallel group design to obtain the same power for a target effect size and type 1 error rate. We calculated the change in adiponectin concentrations from the morning sample (obtained at 07:00 h on the same day) to each of the post-exercise timepoints (11:00, 12:00, and 15:00 h) on each of the test days and used this difference in the statistical analysis. The data were not normally distributed, and log-transformation did not make them so. We therefore decided to analyse differences at each time-point using bootstrapped *t*-tests. The changes (delta value) at each post-exercise timepoint were compared with the changes in concentrations at the corresponding timepoint after REST using paired-samples *t*-tests. The difference at each timepoint is the mean change after MICT or HIIT, compared with the change in the REST condition, for which we report the estimate, corresponding 95% confidence interval (CI), and *p*-value. Since the data were not normally distributed, we used bootstrap with 3000 samples and bias corrected and accelerated CIs. We consider *p*-values < 0.05 as statistically significant and have made no corrections for multiple comparisons due to the exploratory nature of our research question.

## 3 Results

### 3.1 Participants and breast milk adiponectin

**FIGURE 2:**
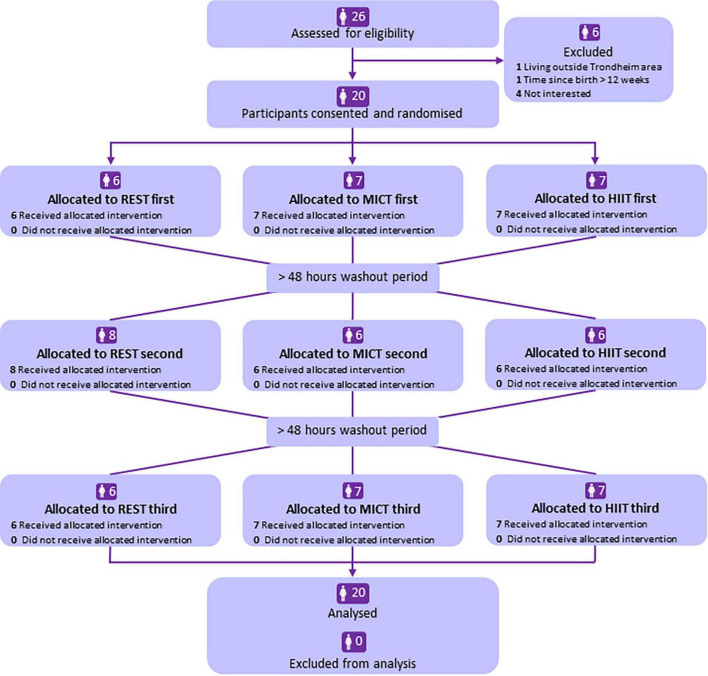
Flowchart of participants in the study. REST, resting condition in the laboratory; MICT, moderate-intensity continuous training; HIIT, high-intensity interval training.

Figure 2 shows the flow of participants across the three conditions. All the 20 participants completed all three conditions and provided breast milk at all the timepoints, thus the total number of breast milk samples analysed was 240. Recruitment of participants started in August 2021 and was completed in May 2022. Table 1 shows participants’ baseline characteristics, according to which condition they completed first (Supplementary Table 1). There were no adverse events. The trial was ended when we had included 20 participants. We detected adiponectin in all breast milk samples, with an average concentration for all 240 samples of 5.2 (SD 2.5) μg/L (range 1.2–12.7 μg/L). The variation within each person (for the 12 samples obtained from each participant) was smaller, with SD 0.9 μg/L (Supplementary Table 2).

**TABLE 1:** Baseline characteristics of participants, by which condition they completed first.

	ALL (*n* = 20)	REST (*n* = 6)	MICT (*n* = 7)	HIIT (*n* = 7)
Age, years	31 (3)	31 (3)	30 (2)	32 (3)
Body mass, kg	72.2 (9.0)	77.8 (9.6)	66.0 (6.2)	73.6 (8.1)
Body mass index, kg/m^2^	26.2 (3.6)	28.3 (3.8)	24.3 (3.0)	26.2 (3.3)
Fat mass, kg	23.4 (8.3)	28.6 (9.3)	18.1 (7.3)	24.2 (5.6)
Peak oxygen uptake, mL⋅kg^–1^⋅min^–1^	39.5 (7.8)	34.7 (5.0)	44.0 (7.9)	38.9 (7.8)
Time since delivery, weeks	8.9 (2.8)	9.1 (2.2)	8.4 (1.9)	9.3 (1.7)
Infant birth weight, g	3652 (422)	3963 (188)	3338 (276)	3700 (493)

Numbers are averages with standard deviations. REST, no exercise; MICT, moderate-intensity continuous training; HIIT, high-intensity interval training.

### 3.2 Acute effects of exercise on breast milk adiponectin concentrations

**FIGURE 3:**
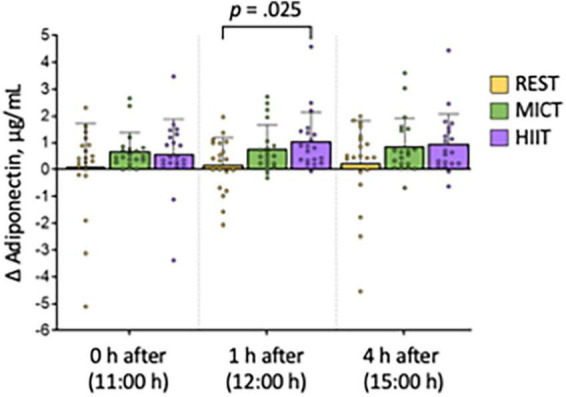
Change in breast milk adiponectin concentrations compared with a sample obtained at 07:00 h at each condition. Bars show average concentrations, error bars show standard deviation, and symbols show individual data. *P*-value is from paired-samples *t*-test and shows the difference at 1 h after high-intensity interval training (HIIT) condition compared with 1 h after no exercise (REST). MICT, moderate-intensity continuous training.

Table 2 shows the concentrations at each time-point for each condition. There were no statistically significant differences between adiponectin concentrations at the first time-point (07:00 h) on the three test days (REST vs. MOD: *p* = 0.143, REST vs. HIIT: *p* = 0.077, MOD vs. HIIT: *p* = 0.831). Compared with the REST condition, adiponectin tended to increase after exercise, statistically significant so only 1 h after HIIT (Figure 3 and Table 3).

**TABLE 2:** Breast milk adiponectin concentrations at different time points in the three conditions.

		Time point			
**Condition**	**Days postpartum**	**07:00 h**	**11:00 h**	**12:00 h**	**15:00 h**
REST	69 (13)	5.2 (3.0) μg/L	5.2 (2.0) μg/L	5.4 (2.6) μg/L	5.4 (2.4) μg/L
MICT	70 (16)	4.6 (2.3) μg/L	5.3 (2.8) μg/L	5.4 (2.8) μg/L	5.5 (2.7) μg/L
HIIT	70 (14)	4.6 (2.2) μg/L	5.1 (2.0) μg/L	5.6 (2.5) μg/L	5.5 (2.5) μg/L

Observed data are presented as descriptive mean with standard deviation (SD) for 20 participants. REST, no exercise; MICT, moderate-intensity continuous training; HIIT, high-intensity interval training.

**TABLE 3:** Changes in adiponectin concentrations (in μg/L) compared with a sample obtained at 07:00 hours (h) at each condition at different time points after moderate-intensity continuous training (MICT) and high-intensity interval training (HIIT), compared with no exercise (REST), with mean difference, corresponding 95% confidence interval (CI), and *p*-values from paired samples *t*-tests.

	Immediately after exercise (11:00 h), compared with no exercise			1 h after exercise (12:00 h), compared with no exercise			4 h after exercise (15:00 h), compared with no exercise		
**Condition**	**Mean difference**	**95% CI**	***p***	**Mean difference**	**95% CI**	***p***	**Mean difference**	**95% CI**	***p***
MICT	0.63	−0.03 to 1.40	0.164	0.60	0.04 to 1.21	0.080	0.64	−0.01 to 1.42	0.158
HIIT	0.55	0.08 to 1.09	0.074	0.89	0.30 to 1.58	0.025	0.75	0.06 to 1.56	0.105

Data are from 20 participants.

### 3.3 Implementation and compliance with exercise protocols and breast milk sampling

The minimum washout period between conditions was, per protocol, 48 h, whereas the actual minimum washout period was 4 days (96 h). The average interval between test-days was 7.3 (SD 2.0) days between the first and second condition, and 6.9 (SD 1.4) days between the second and third condition. Most samples were obtained at the exact time-point prescribed in the protocol, with an average of 2.5 (SD 10.1) min deviation. The largest deviations from the prescribed time-points were at the sampling at 15:00 h (4.7 min, SD 11.4), whereas the samples obtained in the laboratory (at 11:00 h and 12:00 h) deviated by on average <1 min from the protocol. The exercise intensity was 70% (SD 1) of heart rate maximum during MICT and 96% (SD 2) of heart rate maximum during HIIT.

## 4 Discussion

There is scarce research on the acute effects of exercise on breast milk composition and currently few exercise guidelines for lactating people. The most recent recommendations on postpartum physical activity from the American College of Obstetricians and Gynaecologists merely states that *regular aerobic exercise in lactating women has been shown to improve maternal cardiovascular fitness without affecting milk production, composition, or infant growth* (35). These recommendations are based on observational studies (36, 37) and one RCT (38) from the 1990s analysing total concentrations of lipids, protein, lactose, and some minerals in breast milk after exercise.

In our study, we found statistically significant increased (∼ 22%) concentrations of breast milk adiponectin 1 h after high-intensity training (HIIT) in exclusively breastfeeding people, compared with a day with no exercise (REST), with tendencies of elevated concentrations at all other time-points after both moderate intensity continuous training (MICT) and HIIT. These findings suggest that it may take some time before an exercise-induced increase in breast milk adiponectin concentration is evident, and that HIIT is a more potent stimulus for such increase than MICT. On the day that the participants did not exercise (REST), the adiponectin concentrations were quite stable throughout the day (from 07:00 to 15:00 h), suggesting that there is little chronobiological variations in breast milk adiponectin concentrations within this time frame.

There are some limitations to our study, including the relatively small sample size and no control of breastfeeding on the test days. The interval between test days was 7 days for most participants, with 4 days as the shortest (for one participant) and 14 days as the longest (for one participant). We assumed that there would be no order effect and did not control for this in our analysis. There may be differences in adiponectin concentration between foremilk and hindmilk. We asked the participants to collect the breast milk samples before feeding their infants at the set time points, but did not control for the time since last feed.

There could be an alternative explanation for the presented findings of increased adiponectin concentration 1 h after HIIT. Contrary to our expectation, we observed higher average morning concentration at the day of the REST condition despite randomising the order of HIIT, MICT and REST conditions. Thus, the increase we observed at the MICT and HIIT conditions could have been due to regression to the mean. However, circulating adiponectin concentrations (in serum) have shown to be elevated after exercise. While this is the first study on the acute effects of exercise on adiponectin concentrations in breast milk, breast milk adiponectin concentrations have been reported to correlate with concentrations in blood (10). Presuming that there is a correlation between concentrations in these two body fluids, we may compare our findings to studies on acute effect of endurance training on circulating adiponectin levels.

Elevated blood adiponectin concentrations have been observed after different forms of endurance exercise including rowing, running, cycling, and step-aerobic (39–43). In agreement with our findings, Jürimäe and colleagues found that plasma adiponectin concentrations were unchanged immediately after a maximal 6,000 m rowing ergometer test (lasting on average 20 min) in highly trained male rowers but increased by ∼20% after 30 min of recovery (39). In contrast to both Jürimäe and colleagues and our present study, Schön and colleagues reported ∼ 10% elevated serum adiponectin concentrations immediately after a 90-min run at 75–80% of heart rate maximum, followed by a return to baseline concentrations after 60 min of recovery in healthy young individuals (40). There are also studies that suggest no alterations in circulating adiponectin levels acutely after exercise (44–47). The reasons for these diverging findings may include sex-differences, differences in the type of exercise, duration and intensity of exercise, as well as methodological differences in analyses and in time-points of sampling. Our study is the first that has analysed adiponectin concentrations in breast milk after exercise. As studies report that increased concentrations of adiponectin in breast milk may play a role in protection against early rapid weight gain in infancy (15–18), our findings indicate that maternal exercise during lactation can be one of the factors that affect the risk of childhood obesity. If breast milk adiponectin can protect against rapid weight gain in infancy, our results indicate that the best time to breastfeed is around 1 h after high-intensity exercise. Our results should be confirmed in further studies, including sampling of breast milk several hours after an exercise session. We therefore suggest further studies investigating both the immediate influence of a single exercise session on breast milk composition, as well as chronic adaptations with regular exercise training. We also propose that further research should consider the whole breast milk matrix, which is composed of many bio-active components. We envision that more research on the detailed effects of maternal exercise on breast milk composition will provide an evidence-base for more detailed guidelines from the American College of Obstetricians and Gynaecologists and other organisations.

## Data availability statement

The original contributions presented in the study are included in the article/Supplementary material, further inquiries can be directed to the corresponding author.

## Ethics statement

The studies involving humans were approved by the Regionale Komiteer for Medisinsk og Helsefaglig Forskningsetikk (REK). The studies were conducted in accordance with the local legislation and institutional requirements. The participants provided their written informed consent to participate in this study.

## Author contributions

MH: Data curation, Investigation, Methodology, Writing – original draft, Writing – review and editing. GG: Data curation, Formal analysis, Methodology, Writing – review and editing. TM: Conceptualization, Data curation, Formal analysis, Investigation, Methodology, Project administration, Supervision, Writing – original draft, Writing – review and editing.

## References

[1] World Health Organization. *Obesity and overweight.* Geneva: World Health Organization (2021).

[2] Mameli C Mazzantini S Zuccotti G. Nutrition in the First 1000 Days: The Origin of Childhood Obesity. *Int J Environ Res Public Health.* (2016) 13:838. 10.3390/ijerph13090838 PMC5036671 27563917

[3] Derks I Hivert M Rifas-Shiman S Gingras V Young J Jansen P Associations of prenatal exposure to impaired glucose tolerance with eating in the absence of hunger in early adolescence. *Int J Obes.* (2019) 43:1903–13. 10.1038/s41366-018-0296-6 30622313 PMC6614016

[4] Koleva P Bridgman S Kozyrskyj A. The infant gut microbiome: evidence for obesity risk and dietary intervention. *Nutrients.* (2015) 7:2237–60. 25835047 10.3390/nu7042237 PMC4425142

[5] Indrio F Martini S Francavilla R Corvaglia L Cristofori F Mastrolia S Epigenetic matters: the link between early nutrition, microbiome, and long-term health development. *Front Pediatr.* (2017) 5:178. 10.3389/fped.2017.00178 28879172 PMC5572264

[6] Horta B Loret de Mola C Victora C. Long-term consequences of breastfeeding on cholesterol, obesity, systolic blood pressure and type 2 diabetes: a systematic review and meta-analysis. *Acta Paediatr.* (2015) 104:30–7. 10.1111/apa.13133 26192560

[7] Gregg B Ellsworth L Pavela G Shah K Berger P Isganaitis E Bioactive compounds in mothers milk affecting offspring outcomes: a narrative review. *Pediatr Obes.* (2022) 17:e12892. 10.1111/ijpo.12892 35060344 PMC9177518

[8] Moholdt T Stanford K. Exercised breastmilk: a kick-start to prevent childhood obesity? *Trends Endocrinol Metab.* (2023). [Epub ahead of print]. 10.1016/j.tem.2023.08.019 37735048 PMC11005327

[9] Scheja L Heeren J. The endocrine function of adipose tissues in health and cardiometabolic disease. *Nat Rev Endocrinol.* (2019) 15:507–24. 31296970 10.1038/s41574-019-0230-6

[10] Savino F Lupica M Benetti S Petrucci E Liguori S Cordero Di Montezemolo L. Adiponectin in breast milk: relation to serum adiponectin concentration in lactating mothers and their infants. *Acta Paediatr.* (2012) 101:1058–62. 22646778 10.1111/j.1651-2227.2012.02744.x

[11] Ronti T Lupattelli G Mannarino E. The endocrine function of adipose tissue: an update. *Clin Endocrinol.* (2006) 64:355–65. 10.1111/j.1365-2265.2006.02474.x 16584505

[12] Weyer C Funahashi T Tanaka S Hotta K Matsuzawa Y Pratley R Hypoadiponectinemia in obesity and type 2 diabetes: close association with insulin resistance and hyperinsulinemia. *J Clin Endocrinol Metab.* (2001) 86:1930–5. 11344187 10.1210/jcem.86.5.7463

[13] Liu C Feng X Li Q Wang Y Li Q Hua M. Adiponectin, TNF-α and inflammatory cytokines and risk of type 2 diabetes: A systematic review and meta-analysis. *Cytokine.* (2016) 86:100–9. 10.1016/j.cyto.2016.06.028 27498215

[14] Martin L Woo J Geraghty S Altaye M Davidson B Banach W Adiponectin is present in human milk and is associated with maternal factors. *Am J Clin Nutr.* (2006) 83:1106–11. 16685053 10.1093/ajcn/83.5.1106

[15] Young B Levek C Reynolds R Rudolph M MacLean P Hernandez T Bioactive components in human milk are differentially associated with rates of lean and fat mass deposition in infants of mothers with normal vs. elevated BMI. *Pediatr Obes.* (2018) 13:598–606. 10.1111/ijpo.12394 30092608 PMC6390491

[16] Mohamad M Loy S Lim P Wang Y Soo K Mohamed H. Maternal serum and breast milk adiponectin: the association with infant adiposity development. *Int J Environ Res Public Health.* (2018) 15:1250. 10.3390/ijerph15061250 29895806 PMC6025015

[17] Yu X Rong S Sun X Ding G Wan W Zou L Associations of breast milk adiponectin, leptin, insulin and ghrelin with maternal characteristics and early infant growth: a longitudinal study. *Br J Nutr.* (2018) 120:1380–7. 10.1017/S0007114518002933 30375294

[18] Woo J Guerrero M Altaye M Ruiz-Palacios G Martin L Dubert-Ferrandon A Human milk adiponectin is associated with infant growth in two independent cohorts. *Breastfeed Med.* (2009) 4:101–9. 10.1089/bfm.2008.0137 19500050 PMC2779028

[19] Gridneva Z Kugananthan S Rea A Lai C Ward L Murray K Human milk adiponectin and leptin and infant body composition over the first 12 months of lactation. *Nutrients.* (2018) 10:1125. 10.3390/nu10081125 30127292 PMC6115716

[20] Kon I Shilina N Gmoshinskaya M Ivanushkina T. The study of breast milk IGF-1, leptin, ghrelin and adiponectin levels as possible reasons of high weight gain in breast-fed infants. *Ann Nutr Metab.* (2014) 65:317–23. 10.1159/000367998 25402263

[21] Khodabakhshi A Ghayour-Mobarhan M Rooki H Vakili R Hashemy S Mirhafez S Comparative measurement of ghrelin, leptin, adiponectin, EGF and IGF-1 in breast milk of mothers with overweight/obese and normal-weight infants. *Eur J Clin Nutr.* (2015) 69:614–8. 10.1038/ejcn.2014.205 25351650

[22] Chan D Goruk S Becker A Subbarao P Mandhane P Turvey S Adiponectin, leptin and insulin in breast milk: associations with maternal characteristics and infant body composition in the first year of life. *Int J Obes.* (2018) 42:36–43. 10.1038/ijo.2017.189 28925410

[23] Meyer D Brei C Stecher L Much D Brunner S Hauner H. The relationship between breast milk leptin and adiponectin with child body composition from 3 to 5 years: a follow-up study. *Pediatr Obes.* (2017) 12:125–9. 10.1111/ijpo.12192 27863153

[24] Anderson J McKinley K Onugha J Duazo P Chernoff M Quinn E. Lower levels of human milk adiponectin predict offspring weight for age: a study in a lean population of Filipinos. *Matern Child Nutr.* (2016) 12:790–800. 10.1111/mcn.12216 26446289 PMC6860153

[25] Weyermann M Brenner H Rothenbacher D. Adipokines in human milk and risk of overweight in early childhood: a prospective cohort study. *Epidemiology.* (2007) 18:722–9. 18062063 10.1097/ede.0b013e3181567ed4

[26] Samuel T Binia A de Castro C Thakkar S Billeaud C Agosti M Impact of maternal characteristics on human milk oligosaccharide composition over the first 4 months of lactation in a cohort of healthy European mothers. *Sci Rep.* (2019) 9:11767. 10.1038/s41598-019-48337-4 31409852 PMC6692355

[27] Isganaitis E Venditti S Matthews T Lerin C Demerath E Fields D. Maternal obesity and the human milk metabolome: associations with infant body composition and postnatal weight gain. *Am J Clin Nutr.* (2019) 110:111–20. 10.1093/ajcn/nqy334 30968129 PMC6599743

[28] Bravi F Wiens F Decarli A Dal Pont A Agostoni C Ferraroni M. Impact of maternal nutrition on breast-milk composition: a systematic review. *Am J Clin Nutr.* (2016) 104:646–62. 27534637 10.3945/ajcn.115.120881

[29] Zhang L Zhang J Han B Chen C Liu J Sun Z Gestational diabetes mellitus-induced changes in proteomes and glycated/glycosylated proteomes of human colostrum. *J Agric Food Chem.* (2021) 69:10749–59. 10.1021/acs.jafc.1c03791 34474557

[30] Seferovic M Mohammad M Pace R Engevik M Versalovic J Bode L Maternal diet alters human milk oligosaccharide composition with implications for the milk metagenome. *Sci Rep.* (2020) 10:22092. 10.1038/s41598-020-79022-6 33328537 PMC7745035

[31] Khalafi M Symonds M. The impact of high-intensity interval training on inflammatory markers in metabolic disorders: A meta-analysis. *Scand J Med Sci Sports.* (2020) 30:2020–36. 10.1111/sms.13754 32585734

[32] Yu N Ruan Y Gao X Sun J. Systematic review and meta-analysis of randomized, controlled trials on the effect of exercise on serum leptin and adiponectin in overweight and obese individuals. *Horm Metab Res.* (2017) 49:164–73. 10.1055/s-0042-121605 28249299

[33] Berglund I Soras S Relling B Lundgren K Kiel I Moholdt T. The relationship between maximum heart rate in a cardiorespiratory fitness test and in a maximum heart rate test. *J Sci Med Sport.* (2019) 22:607–10. 10.1016/j.jsams.2018.11.018 30527685

[34] Rognmo O Hetland E Helgerud J Hoff J Slordahl S. High intensity aerobic interval exercise is superior to moderate intensity exercise for increasing aerobic capacity in patients with coronary artery disease. *Eur J Cardiovasc Prev Rehabil.* (2004) 11:216–22. 15179103 10.1097/01.hjr.0000131677.96762.0c

[35] American College of Obstetricians and Gynecologists. Physical activity and exercise during pregnancy and the postpartum period: ACOG committee opinion, number 804. *Obstet Gynecol.* (2020) 135:e178–88. 32217980 10.1097/AOG.0000000000003772

[36] Lovelady C Lonnerdal B Dewey K. Lactation performance of exercising women. *Am J Clin Nutr.* (1990) 52:103–9. 2360539 10.1093/ajcn/52.1.103

[37] Fly A Uhlin K Wallace J. Major mineral concentrations in human milk do not change after maximal exercise testing. *Am J Clin Nutr.* (1998) 68:345–9. 9701192 10.1093/ajcn/68.2.345

[38] Dewey K Lovelady C Nommsen-Rivers L McCrory M Lönnerdal B. A randomized study of the effects of aerobic exercise by lactating women on breast-milk volume and composition. *N Engl J Med.* (1994) 330:449–53. 8289849 10.1056/NEJM199402173300701

[39] Jürimäe J Purge P Jürimäe T. Adiponectin is altered after maximal exercise in highly trained male rowers. *Eur J Appl Physiol.* (2005) 93:502–5. 10.1007/s00421-004-1238-7 15618990

[40] Schön M Kovaničová Z Košutzká Z Nemec M Tomková M Jacková L Effects of running on adiponectin, insulin and cytokines in cerebrospinal fluid in healthy young individuals. *Sci Rep.* (2019) 9:1959. 10.1038/s41598-018-38201-2 30760755 PMC6374465

[41] Saunders T Palombella A McGuire K Janiszewski P Després J Ross R. Acute exercise increases adiponectin levels in abdominally obese men. *J Nutr Metab.* (2012) 2012:148729. 10.1155/2012/148729 PMC3369484 22701167

[42] Öztürk G Kaya O Gürel E Palabiyik OK Süt N Öztürk L Acute supramaximal exercise-induced adiponectin increase in healthy volunteers: involvement of natriuretic peptides. *Adipobiology.* (2016) 8:39–45.

[43] Sari I Habipoğlu S Seydel G Erşan S Güntürk I. The effect of acute step-aerobic exercise on adiponectin and leptin levels in premenopausal women. *J Sports Med Phys Fitness.* (2021) 61:725–31. 10.23736/S0022-4707.20.11297-0 33146490

[44] Jamurtas A Theocharis V Koukoulis G Stakias N Fatouros I Kouretas D The effects of acute exercise on serum adiponectin and resistin levels and their relation to insulin sensitivity in overweight males. *Eur J Appl Physiol.* (2006) 97:122–6. 10.1007/s00421-006-0169-x 16525810

[45] Ferguson M White L McCoy S Kim H Petty T Wilsey J. Plasma adiponectin response to acute exercise in healthy subjects. *Eur J Appl Physiol.* (2004) 9:324–9. 10.1007/s00421-003-0985-1 14586663

[46] Bouassida A Lakhdar N Benaissa N Mejri S Zaouali M Zbidi A Adiponectin responses to acute moderate and heavy exercises in overweight middle aged subjects. *J Sports Med Phys Fitness.* (2010) 50:330–5. 20842095

[47] Antunes B Rossi F Oyama L Rosa-Neto J Lira F. Exercise intensity and physical fitness modulate lipoproteins profile during acute aerobic exercise session. *Sci Rep.* (2020) 10:4160. 10.1038/s41598-020-61039-6 32139762 PMC7058045

