# Supplementary material for: High-intensity exercise increases breast milk adiponectin concentrations: a randomised cross-over study
Source: Front Nutr. 2023 Dec 18;10:1275508. doi: 10.3389/fnut.2023.1275508 (PMC10757973; doi:10.3389/fnut.2023.1275508)
Supplement: Supplementary file 1 [file Data_Sheet_1.pdf]

Supplementary Information

Supplementary Table 1. Participant data.

ID	Age, years	Weeks post-partum	Infant birth-weight, G	Sequence	Weight, kg	Height, cm	Muscle mass, kg	Fat mass, kg	BMI, kg/m^2	VO_2peak, mg/kg/min	HR max, beats/min	Days between visit 1 and 2	Days between visit 2 and 3	Exercise intensity HIIT, % of HR max	Exercise intensity MICT, % of HR max
1	29,2	8,3	4510	H-M-R	79,3	166	26,8	30,8	28,8	33,86	193	7,0	7,0	96%	69%
2	30,6	8,6	3775	R-H-M	83,3	160	26,3	35,8	32,5	34,25	200	7,0	7,0	94%	70%
3	30,9	8,4	3920	R-M-H	93,1	167,5	28,4	42,2	33,4	31,5	194	7,0	7,0	97%	69%
4	30,4	10,9	2980	H-R-M	70,2	174,7	25,6	23,3	23	38,16	192	7,0	7,0	96%	73%
5	32,8	6,9	2945	M-R-H	76,4	160	25	31,2	29,8	33,6	201	7,0	8,0	97%	70%
6	27,4	12,4	3910	R-M-H	73,6	167	27,7	23,4	26,4	40,07	196	7,0	7,0	96%	70%
7	30,2	7,3	3785	R-H-M	72,4	166	25,5	25,7	26,3	29,99	182	7,0	6,0	99%	71%
8	29,3	8,4	3150	M-H-R	64,3	166	28,1	13,7	23,3	48,27	181	7,0	7,0	97%	71%
9	34,1	11,3	3210	M-R-H	65,3	166	26,8	16,9	23,7	56,6	186	7,0	7,0	94%	68%
10	32,0	7,3	3985	H-M-R	66,6	161,5	26,1	19,7	25,5	37,88	197	10,0	6,0	95%	72%
11	27,8	8,6	3470	H-M-R	64,4	166	24,9	19	23,4	52,31	193	7,0	9,0	95%	71%
12	29,5	11,0	3620	M-H-R	66,7	171	31,4	10,9	22,8	45,82	186	4,0	5,0	97%	70%
13	29,5	7,1	3260	M-R-H	55,2	165	24,2	11,1	20,3	47,6	183	7,0	7,0	95%	68%
14	32,3	7,9	3495	H-R-M	68,3	170	27,5	17,8	23,6	45,58	185	7,0	7,0	96%	70%
15	27,6	7,1	3730	M-R-H	66,2	166	23,6	22,8	24	39,22	184	9,0	4,0	97%	72%
16	35,7	6,6	4190	R-M-H	65,7	167	27,7	16,1	23,6	41,66	187	7,0	7,0	97%	72%
17	38,4	11,3	3960	H-R-M	84,8	163	31,1	29,9	31,9	36,43	170	4,0	6,0	91%	70%
18	30,5	11,0	4195	R-H-M	78,9	169	27,6	28,5	27,6	30,87	169	14,0	6,0	95%	68%
19	33,7	11,1	3500	H-R-M	81,5	174	28,9	29	26,9	28,39	193	7,0	7,0	91%	70%
20	29,8	7,0	3450	M-R-H	67,9	161	26,7	19,9	26,2	37,17	187	6,0	11,0	96%	71%

BMI = Body mass index, VO_2peak = Peak oxygen uptake, HR max = Maximum heart rate, HIIT = High-intensity interval training, MICT = Moderate intensity continuous training.

Supplementary Table 2. Adiponectin data, in µg/L

ID	**REST 07:00 h**	**REST 11:00 h**	**REST 12:00 h**	**REST 15:00 h**	**MICT 07:00 h**	**MICT 11:00 h**	**MICT 12:00 h**	**MICT 15:00 h**	**HIIT 07:00 h**	**HIIT 11:00 h**	**HIIT 12:00 h**	**HIIT 15:00 h**
1	4,005	4,692	4,99	5,993	4,024	4,063	4,906	4,736	3,345	6,817	7,92	7,781
2	8,093	8,323	9,21	9,689	7,371	7,739	7,536	8,883	8,678	10,172	9,042	8,05
3	11,46	8,327	9,401	6,921	10,254	12,263	12,731	9,572	6,753	5,639	8,958	7,881
4	6,901	4,997	5,333	4,855	4,793	5,342	5,119	5,119	5,027	6,695	5,201	4,949
5	1,343	2,511	2,381	2,188	1,152	1,497	1,638	1,832	1,502	1,974	2,183	2,736
6	2,967	3,213	2,964	4,202	2,252	2,706	2,892	2,458	3,625	4,089	3,803	3,846
7	6,932	6,705	8,902	8,765	7,66	8,858	8,712	9,611	6,943	7,179	8,052	8,66
8	3,119	3,52	3,438	4,059	4,631	5,107	4,857	4,675	2,776	2,911	3,106	2,989
9	4,925	5,808	4,131	4,367	2,715	3,516	3,216	2,765	2,454	2,705	2,957	3,177
10	2,573	3,97	3,099	3,065	2,983	3,361	3,047	3,009	1,777	3,236	3,134	3,179
11	4,185	6,494	4,56	4,632	4,156	4,844	6,106	4,799	3,998	5,193	4,886	4,556
12	6,525	6,622	5,846	6,949	6,518	6,956	6,988	7,065	5,674	6,664	6,969	7,227
13	2,674	3,168	3,08	3,181	2,484	2,757	2,7	2,912	3,171	4,082	4,594	3,317
14	4,373	4,178	4,374	4,287	4,379	4,391	4,064	4,426	3,981	4,142	4,209	4,081
15	2,071	2,079	2,049	2,064	2,214	2,384	2,082	2,415	2,384	2,728	2,314	2,653
16	2,569	3,481	3,546	3,012	2,542	3,397	3,33	3,285	3,181	4,077	4,038	3,884
17	3,901	5,498	5,263	5,633	4,226	4,98	4,951	5,668	3,806	5,115	5,35	4,869
18	11,68	6,571	10,697	9,197	5,497	5,501	5,498	9,096	8,794	5,401	11,277	11,249
19	8,445	8,743	8,362	9,555	7,128	7,837	9,357	8,713	6,88	7,155	7,683	8,657
20	4,843	5,264	5,496	5,222	5,59	8,246	8,304	8,626	6,494	6,832	6,866	6,801

REST = No activity, MICT = Moderate intensity continuous training, HIIT = High-intensity interval training.
